# Dose Assessment of Cefquinome by Pharmacokinetic/Pharmacodynamic Modeling in Mouse Model of *Staphylococcus aureus* Mastitis

**DOI:** 10.3389/fmicb.2016.01595

**Published:** 2016-10-07

**Authors:** Yang Yu, Yu-Feng Zhou, Xiao Li, Mei-Ren Chen, Gui-Lin Qiao, Jian Sun, Xiao-Ping Liao, Ya-Hong Liu

**Affiliations:** ^1^National Risk Assessment Laboratory for Antimicrobial Resistance of Animal Original Bacteria, South China Agricultural UniversityGuangzhou, China; ^2^Guangdong Provincial Key Laboratory of Veterinary Pharmaceutics, Development and Safety Evaluation, South China Agricultural UniversityGuangzhou, China; ^3^Defense Threat Reduction Agency, Fort BelvoirVA, USA; ^4^College of Veterinary Medicine, National Reference Laboratory of Veterinary Drug Residues, South China Agricultural UniversityGuangzhou, China

**Keywords:** dose assessment, PK/PD, Monte Carlo simulation, cefquinome, mastitis

## Abstract

This work aimed to characterize the mammary gland pharmacokinetics of cefquinome after an intramammary administration and integrate pharmacokinetic/pharmacodynamic model. The pharmacokinetic profiles of cefquinome in gland tissue were measured using high performance liquid chromatograph. Therapeutic regimens covered various dosages ranging from 25 to 800 μg/gland and multiple dosing intervals of 8, 12, and 24 h. The *in vivo* bacterial killing activity elevated when dosage increased or when dosing intervals were shortened. The best antibacterial effect was demonstrated by a mean 1.5 log_10_CFU/gland visible count reduction. On the other hand, the results showed that the percentage of time duration of drug concentration exceeding the MIC during a dose interval (%T > MIC) was generally 100% because of the influence of drug distribution caused by the blood-milk barrier. Therefore, pharmacokinetic/pharmacodynamic parameter of the ratio of area under the concentration-time curve over 24 h to the MIC (AUC_0-24_/MIC) was used to describe the efficacy of cefquinome instead of %T > MIC. When the magnitude of AUC_0-24_/MIC exceeding 16571.55 h⋅mL/g, considerable activity of about 1.5 log_10_CFU/g gland bacterial count reduction was observed *in vivo*. Based on the Monte Carlo simulation, the clinical recommended regimen of three infusions of 75 mg per quarter every 12 h can achieve a 76.67% cure rate in clinical treatment of bovine mastitis caused by *Staphylococcus aureus* infection.

## Introduction

*Staphylococcus aureus* is a common Gram-positive bacterium that frequently causes a variety of infections in humans and animals and is the primary pathogen responsible for bovine mastitis. *S. aureus* mastitis can lead to significant economic loss to the dairy industry due to the deterioration of milk quality, veterinary medicine expenses, and prohibitive labor costs (Gruet et al., 2001). According to the clinical features, intramammary infection (IMI) is classified as clinical and subclinical mastitis. Clinical mastitis is acute and severe and may cause cow’s death. While subclinical mastitis is generally not lethal, but can lead to huge financial losses. *S. aureus* can be isolated from the mammary gland (MG) tissue of all forms of mastitis because these organisms are capable of hiding in host phagocytes and mammary epithelial cell to avoid antibiotic effect (Hebert et al., 2000).

Cefquinome is a semisynthetic β-lactam antibiotic and fourth-generation cephalosporin developed for use in veterinary medicine. It is stable to common plasmid- and chromosomally mediated β-lactamases. For example, cefquinome showed higher ability to treat the equine infection than penicillin G and gentamicin (Widmer et al., 2009). When using cefquinome in treatment of IMI diseases, a considerable therapeutic effect is found: the clinical persistence and recurrence of bovine mastitis are reduced during lactation; and the treated cows are less likely to develop clinical mastitis in the dry period (Bradley et al., 2011; Swinkels et al., 2013). Cefquinome can be administered by a parenteral route, intramammary infusion, or parenteral injection combined with intramammary infusion in China. The intramammary treatment may acquire a higher cure rate compared with systemic administration (Shpigel et al., 2006).

With regard to optimization of therapy regimen, pharmacokinetic and pharmacodynamic (PK/PD) model is an advanced approach concurrently analyzing the time course and the antibacterial effectiveness of a drug. The PK/PD analysis may further elucidate an inadequate daily dose or extended dosing interval accompanied under traditional dosing regimen determination. In our previous work, the PK/PD characteristics, especially in the blood, were studied against the *S. aureus* in a mouse mastitis model following an intramammary administration (Yu et al., 2016). However, we wondered how the drug concentrations would be in local MG and if it might be better to use MG PK data to optimize the dosage, given that in some organs (like brain or MG) drug distribution become much more complex due to the special anatomic structures or transport barriers.

The objective of this work was to characterize the PK of cefquinome in MG tissue after an intramammary infusion and integrate PK/PD model of MG tissue. In addition, analysis of surrogate PK/PD indexes required for different levels of antibacterial activity was estimated using the inhibitory sigmoid *E_max_* PD model. Furthermore, we aimed to extrapolate the PK/PD profiles to bovine mastitis treatment and assess the clinical therapeutic regimen using Monte Carlo simulation.

## Materials and Methods

### Bacterial Strains, Reagents, and Animals

*SStaphylococcus aureus* isolates from bovine mastitis was the same population reported by our previous work (Yu et al., 2016), of which the MIC_90_ was 0.5 μg/ml. A similar sensitivity of these isolates to cefquinome was supported by determining the time-killing curves *in vitro* (**Supplementary Figure S1**). Therefore, isolate JP41 of MIC equally to 0.5 μg/ml was chosen randomly for the succeeding trials. The stock solution of cefquinome (Qilu Animal Health Products CO., Ltd, Shandong, China) was prepared in sterile water at 40,000 μg/mL and stored at -20°C until use.

Lactating mice (purchased from Vital River Laboratories, Beijing, China) with body weight of 35–45 g, breeding in a special-pathogen-free (SPF) environment with a 12:12 light: dark circle were used in this study. Experiments were conducted on the L4 (fourth on the left) and R4 (fourth on the right) abdominal glands, which have the biggest size among the whole five pairs of mouse glands and can be harvested easily. The animal studies were approved by the Animal Use and Care Committee of South China Agricultural University. During the *in vivo* procedures, guidelines of American Association for Accreditation of Laboratory Animal Care (Institute of Laboratory Animal Research, Commission on Life Sciences, National Research Council, 1996) had been properly respected.

### Calculation of PK in MG Tissue

Firstly, three healthy CD-1 lactating mice were employed to evaluate the influence of drug distribution on the concentrations of L4 and R4 glands. In brief, 1–2 h following removal of 10–12 day-old offspring, lactating mice were intramammary administrated to just one abdominal gland (L4 or R4). Through a small cut under a teat, 100 μL of cefquinome (1000 μg/mL) was injected into the exposed udder canal using a 32-gage blunt needle. Both the processed and non-treated glands were harvested at time points of 0.08, 0.17, 0.25, 0.5, 0.75, 1, 2, 4, 8, 12, and 24 h after administration. Then drug concentrations in L4 and R4 abdominal glands were measured.

Secondly, MG tissue PK study was performed at a single dose of 25, 50, 100, or 200 μg/gland intramammary infusion into both the L4 and R4 glands (each gland as an individual), 5 mice a group (i.e., *n* = 10 for glands). The administrative procedure was described above. The R4 and L4 MG samples were harvested at 0.08, 0.17, 0.25, 0.5, 0.75, 1, 2, 4, 6, 8, 12, and 24 h after administration.

All the MG samples were processed and analyzed for cefquinome concentrations, and the extracting method and high performance liquid chromatograph (HPLC) condition were described below.

### Determination of Cefquinome in MG Tissue

The gland tissues were homogenized and processed based on the previous report with some modification (Sørensen and Snor, 2000). Briefly, a weight of 0.5 g tissue sample was transferred to a 15 mL polypropylene centrifuge tube and a volume of 5 mL acetonitrile was added. The mixture was shaken vigorously for 2 min using Lab dancer machine (IKA, German) and then centrifuged (Thermo Fisher Scientific, USA) at 5,000 *g* for 10 min. The supernatant was removed and tissue in the bottom was extracted once more again with 2 mL acetonitrile. The supernatant twice extracted was evaporated under a gentle steam of nitrogen (MIULAB, Hangzhou, China) at 38–40°C. The extract was diluted with 5 ml of water and cleaned up by tC_18_ solid-phase extraction (SPE) cartridge (Waters CO., USA). The analytes were eluted with 2 mL acetonitrile and evaporated under a stream of nitrogen at 38–40°C. The pellet was redissolved in 1 mL ultrapure water and filtered through a 0.22 μm syringe filter for HPLC analysis (Ultimate 3000, Dionex), equipped with a RP18 column (4.6 mm × 150 mm, 5 μm; Waters Co., USA). The injection volume was 50 μl, and column temperature was maintained at 30°C. The mobile phase consisted of acetonitrile and 5 mM ammonium acetate containing 0.1% formic acid (v/v, 13/87) provided as an isocratic elution with a flow rate of 250 μl/min. The total run time was 7 min.

The extraction recovery (R_E_) and coefficient of variation (CV) of intra-assay and inter-assay were calculated. Samples of 10, 20, and 50 μg/gland were prepared by adding the standard work solution directly onto the blank gland tissue. After a 30-min incubation for mixing, samples were homogenized, processed, and tested by HPLC as described above. Triple parallels of each concentration for one trial were performed three times totally. The formulas of R_E_ and CV were as follows:

(1) $$R_{\text{E}}(\%)=\frac{C}{C_{\text{a}}}\times 100\%$$

(2) $$\text{CV}(\%)=\frac{\sqrt{\sum_{i=1}^{n}\left(x_{i}-x^{-}\right)/(n-1)}}{x^{-}}\times 100\%$$

Where *C* is calculated drug concentration and *C_a_* is added concentraion; *n* represents for the repeater, x^-^ is average value of concentration.

### Design of PD Experiments

Three CD-1 lactating mice were employed for each condition of treatment using the mouse model of *S. aureus* mastitis (Brouillette et al., 2005; Yu et al., 2016). Totally, 21 therapeutic regimens were investigated in this work. The treatment doses ranged from 25 to 800 μg per gland, and the dosing intervals were 8, 12, and 24 h, respectively. An overnight culture of *S. aureus* JP41 isolates in BHI broth was injected in mice MG after an appropriate dilution. When bacterial counts reaching 7 log_10_CFU/gland in gland tissue (∼9 h incubation after inoculation), cefquinome was administrated to L4 and R4 glands simultaneously and at the corresponding dosing intervals during the 24 h experimental circle. After 24 h treatment, three mice in each group were euthanized for colony count determination (i.e., *n* = 6 for glands). The mice in non-treated control group were tested before the intramammary administration and after 24 h.

### PK/PD Analysis

The cefquinome PK of gland tissue was analyzed using the non-compartment model and one-compartment with non-absorption model, respectively, by WinNonlin software (version 5.2.1; Pharsight, USA). The surrogate markers of antibacterial efficacy, including the ratio of area under the concentration-time curve over 24 h to the MIC (AUC_0-24_/MIC), the percentage of time duration of drug concentration exceeding the MIC during a dose interval (%T > MIC) and the ratio of peak concentration divided by the MIC (C_max_/MIC), were formulated by using *in vitro* MIC_90_ values in broth and *in vivo* PK parameters obtained after intramammary administration of cefquinome. The units of C_max_ and AUC in gland tissue were ug/g and hr⋅ug/g, respectively. The PK/PD parameters of the entire dosing regimens were obtained by extrapolation of the PK profiles determined above.

The antimicrobial effect of cefquinome was analyzed applying the sigmoid *E_max_* model of inhibitory effect, as previously reported (Zhao et al., 2014), which is defined as

(3) $$E=E_{\max}-\frac{(E_{\text{max}}-E_{\text{o}})\times C_{\text{e}}^{N}}{EC_{50}^{N}+C_{\text{e}}^{N}}$$

where *E* is the antibacterial effect, measured as the change in the bacterial counts (log_10_CFU/g gland) in the gland sample after 24 h of treatment compared to the initial colony counts; *E_max_* is the Δlog_10_CFU/g gland in the drug-free control sample; *E_0_* is the Δlog_10_CFU_24__h_/g gland in the test sample containing cefquinome, when the maximum antibacterial effect was achieved; *C_e_* is the PK/PD index (AUC_0-24_/MIC, C_max_/MIC or %T > MIC for gland tissue); *EC_50_* is the value of PK/PD index of drug producing 50% of the maximum antibacterial effect; and N is the Hill coefficient, which describes the steepness of the concentration-effect curve.

### Monte Carlo Simulation

Based on a previous PK study (Li et al., 2014), MIC data (Yu et al., 2016), and the value of PK/PD target magnitude in this work, simulation with Crystal Ball Professional V7.2.2 software was performed for 5,000 sessions. The probability of target attainments (PTAs) of the clinically recommended dose were investigated here. Based on the previous PK study of cefquinome in cows, AUC of milk sample was assumed to be normally distributed in the form of mean values and standard deviation of 4890.19 ± 1906.98 h⋅μg/mL (Li et al., 2014). The probability density functions (PDF) of MIC was custom defined that frequencies of MIC being 0.25 and 0.5 μg/ml were 0.32 and 0.68, respectively, according to our previous study (Yu et al., 2016). The target values of AUC/MIC were obtained in this work, of which 1- or 1.5-log-unit bacterial reduction can be achieved *in vivo*.

To assess therapeutic effect of cefquinome, the PDF of AUC and MIC were the two main components for Monte Carlo simulation. Random sampling the stochastic variable of specified PDFs, thousands of estimation of AUC/MIC and its range of probability will be attained. Then a target value of AUC/MIC was set to calculate the attainment rate of the corresponding dosing regimen, which is defined as the PTA.

## Results

### PK Profiles in Gland Tissues after Intramammary Administration

The limit of quantification (LOQ) was 50 ng/mL and the limit of detection (LOD) was 10 ng/mL in MG tissue. The R_E_s for 10, 20, and 50 μg/gland were 95.43 ± 2.16%, 87.86 ± 4.99% and 73.65 ± 3.22%, respectively. In addition, the CVs of intra-assay and inter-assay are presented in Supplementary Table S1.

No adverse effects, like death of stress, acute death, depression, and abnormal behavior, were observed after intramammary administration. **Supplementary Figure S2** shows the concentration-time curves of both glands, following cefquinome administration into only one MG. The level of cefquinome in the non-treated gland was over 100 times lower than in the administrated one and as low as the LOQ, suggesting that the influence of drug administration to one side of gland on the concentration of the other non-dosed side should be negligible in the same subject. Therefore, we should be able to treat an individual gland as an independent study sample in the study design without concerning the inter-gland drug transfer impact.

**FIGURE 1:**
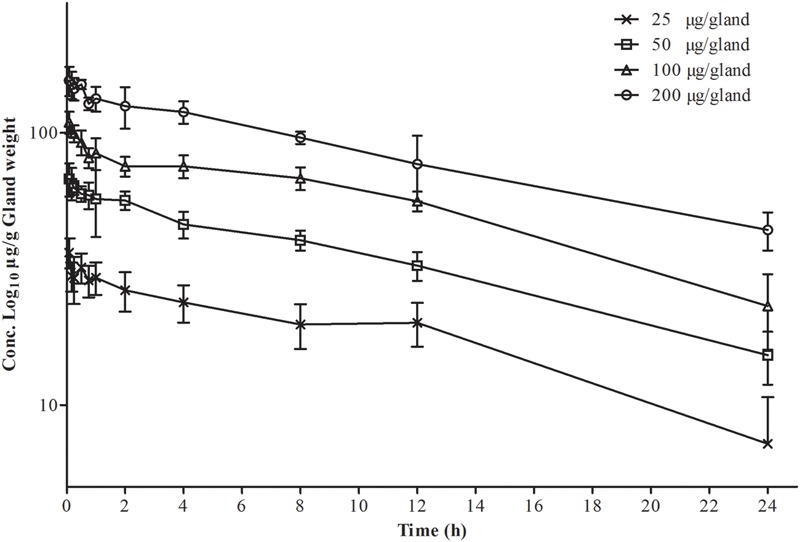
**Semi-logarithmic plot of gland tissue concentration versus time of cefquinome in CD-1 mice following an intramammary administration dose of 25, 50, 100, 200 μg/gland.** Each time point represents the arithmetic mean of five mice (for gland tissue, *n* = 10).

Logarithmic concentration-time plots of the MG tissue data after intramammary administration on both sides of the fourth gland are displayed in **Figure 1**. Profiles of PK in gland tissue are presented in **Table 1**, being analyzed via non-compartmental and one-compartmental models, respectively. The median correlation coefficient (*R*^2^) of four concentration-time curves was equal to 0.93 for the non-compartment model and 0.94 for the one-compartment model. Elimination half-life *t*_1/2-MG_ of 12.44 ± 0.81 and 12.66 ± 0.69 h was calculated, respectively. The AUC_0-24_, being analyzed via the one-compartment with non-absorption model, was slightly higher than the non-compartment model. The mean residence time (MRT_MG_) determined by non-compartment model was 9.09 ± 2.31 h. The eliminating pattern of cefquinome and the comparable value of PK parameters obtained by those two Winnonlin models demonstrated that the PK characteristic in glandular tissue was eliminated exponentially, or following first-order kinetics. Therefore, PK features of multiple dosing were extrapolated from the values obtained in the study described above.

**Table 1:** Pharmacokinetics of cefquinome in MG tissue after a single intramammary administration at dose of 25, 50, 100, and 200 μg per gland to CD-1 mice and analyzed by non-compartment model and one-compartment model, respectively.

Variable(units)	Intramammary administration dose (μg/gland) (*n* = 10)				
	25	50	100	200	Mean ± SD
**Non-compartment model**					
T_1/2-MG_ (h)	12.02	11.79	12.34	13.62	12.44 ± 0.81
AUC_0-24-MG_ (h⋅μg/g)	439.41	827.80	1334.46	2017.92	
MRT_MG_ (h)	9.08	8.94	9.08	10.00	9.09 ± 0.14
*R*^2^ (%)	0.8955	0.9793	0.8961	0.9665	0.93 ± 0.045
**One-compartment model**					
T_1/2-MG_ (h)	12.95	11.63	13.06	13	12.66 ± 0.69
AUC_0-24-MG_ (h⋅μg/g)	585.88	1064.86	1804.91	2756.41	
C_max-MG_ (μg/g)	31.37	63.49	95.8	146.92	
*R*^2^ (%)	0.9095	0.9763	0.8949	0.9637	0.94 ± 0.043

*T_1/2_, elimination half-live representing the procedure that cefquinome transfers from gland tissue to blood; *R*^2^, correlation of concentration-time curves, _MG_, mammary gland. All the parameters are analyzed using five mice (10 glands) average curve data.*

### PD Evaluation of Various Dosing Regimens

**FIGURE 2:**
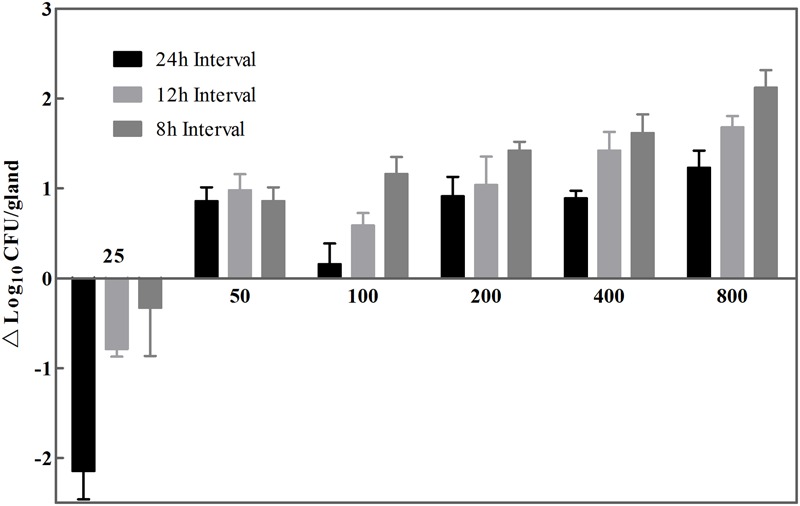
**Survived strains size of *S. aureus* wild isolate JP41 after treating with cefquinome at *t* = 24 h.** Eighteen dose regimens comprised seven dose levels (25, 50, 100, 200, 400, and 800 μg/gland and three intervals (every 8, 12, and 24 h). A mean value of 7.28 log_10_CFU/gland of initial bacterial load was represented as dotted line (*n* = 6 for glands). The limit of detection was shown as full line.

The therapeutic activity of cefquinome was evaluated by bacterial counts (log_10_CFU/gland) at *t* = 24 h in mouse model of *S. aureus* mastitis. **Figure 2** shows antibacterial effects against isolate JP41 of 18 therapeutic regimens, with dosages ranged from 25 to 800 μg/gland at three dosing intervals of 8, 12, and 24 h, respectively. The minimal dose amounts to prevent the microbial growth with 24, 12, and 8 h dosing intervals were 100, 50, and 25 μg/gland, respectively. Among the single daily dose groups, the greatest antibacterial effect was 1.23 log-unit reduction of bacterial counts when giving the largest dose of 800 μg/gland. However, when the dose level was exceeding 400 μg/gland and with 8 or 12 h dosing intervals at the same time, a better antibacterial activity was observed with 1.5 log_10_CFU/gland reductions or more. As the dose increased and the dosing intervals shorten, the antibacterial effectiveness of cefquinome was elevated *in vivo*, exhibiting a declining trend of survival cells by the end of experimental circle (**Figure 2**).

### Integration of PK/PD Parameters

**FIGURE 3:**
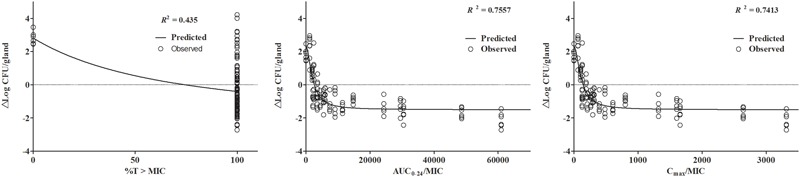
**Relationship between PK/PD parameters in gland tissue and drug killing effectiveness (^Δ^log_10_ CFU/gland) of *S. aureus* JP41 analyzing by the sigmoid model.** The dots represent the antimicrobial effectiveness of cefquinome (*E* = final log-unit – initial log-unit) and the lines denoting the predicted value of *E* which is simulated from the Winnonlin software. The correlation of observed and predicted *E* value was quite low in %T > MIC section because of the distribution of %T > MIC (either 100 or 0%), which is not appropriate for PK/PD integration.

Simulation of PK/PD data against isolate JP41 demonstrated a correlation coefficient (R^2^) of 0.435, 0.7557 and 0.7413 for %T > MIC, AUC_0-24_/MIC, and C_max_/MIC respectively (**Figure 3**). However, cefquinome concentrations in gland tissue were maintained above the MIC of 0.5 μg/mL all the time, so the %T > MIC_90_ was 100% during the 24 h experimental circle. Therefore, the relationship between gland tissue PK and PD activity was reflected by the PK/PD parameter of AUC_0-24_/MIC_90_ (h⋅mL/g) instead. According to the sigmoid model, the best killing activity (*E_0_*) was about 1.5 log_10_CFU/gland bacterial count reductions using either AUC_0-24_/MIC or C_max_/MIC analysis. The *E_max_* and *EC_50_* were 2.03 log_10_CFU/gland and 2483.88 h⋅mL/g for AUC_0-24_/MIC. The calculated ratios of AUC_0-24_/MIC to provide 1 and 1.5 log_10_ CFU/g gland bacterial load drops were 4714.72 and 16571.55 h⋅mL/g (**Table 2**).

**Table 2:** The AUC_0-24_/MIC in MG tissue against *S. aureus* JP41 using the inhibitory form *E_max_* sigmoid model after intramammary administration.

Parameter	AUC_0-24_/MIC
Log *E*_max_ (log_10_CFU/gland)	2.03 ± 0.23
Log *E*_0_ (log_10_CFU/gland)	-1.98 ± 0.20
Log *E*_max_ – Log *E*_0_ (log_10_CFU/gland)	4.01 ± 0.34
EC_50_ (h⋅mL/g)	2483.88 ± 405.55
For bacteriostatic action	2557.56 ± 49.55
For 1 log_10_CFU/gland reduction	4714.72 ± 8.49
For 1.5 log10CFU/gland reduction	16571.55 ± 49.57
Slope (*N*)	1.05 ± 0.17

*The units of AUC_0-24_/MIC are and h⋅mL/g, respectively. PK/PD data for %T > MIC were not available. Bacteriostatic action means no change about bacterial colony counts after 24 h incubation.*

### Monte Carlo Simulation

**FIGURE 4:**
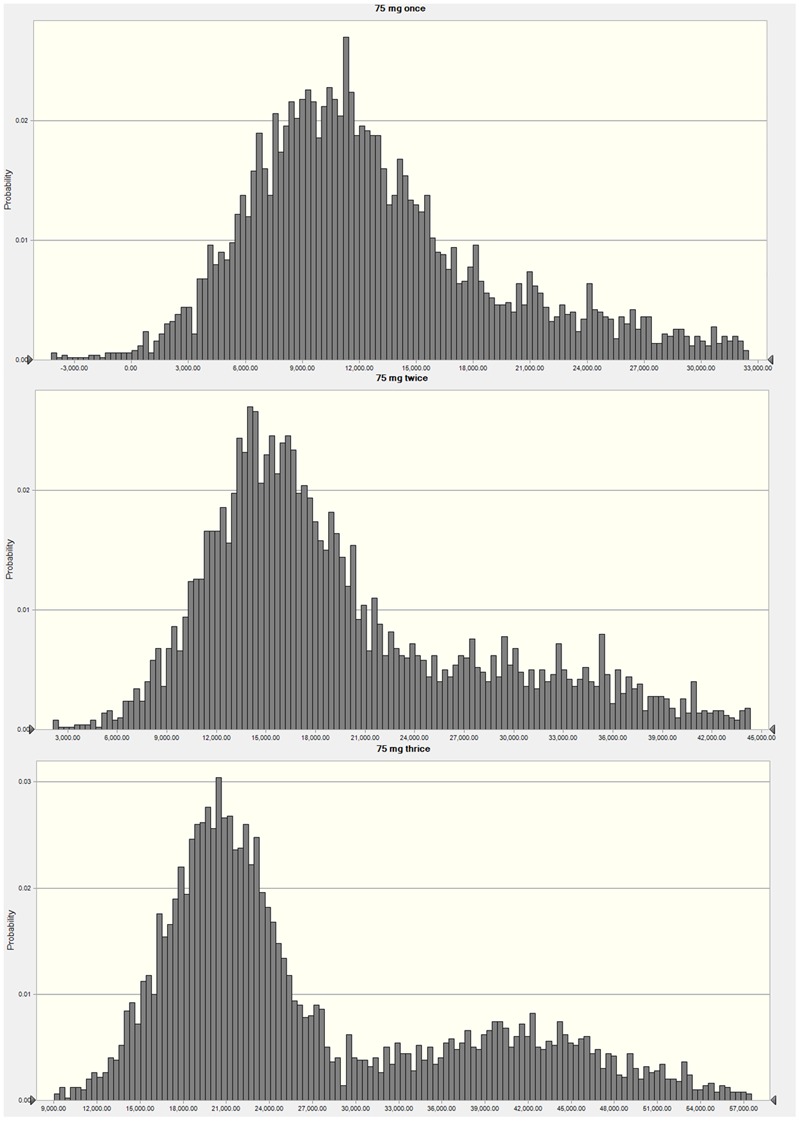
**Frequency distribution plots of AUC/MIC analyzed by Monte Carlo simulation mimicking cefquinome dosing regimens of 75 mg intramammary infusion once, twice, and three times**.

**Figure 4** exhibits the AUC/MIC distribution of three regimens of 75 mg once, twice and thrice doses. When the target value of AUC/MIC was set for 1-log-unit decrease, an over 90% PTAs was calculated following different regimens. However, values of PTAs for 1.5-log-unit reduction were much lower, which were 23.12, 60.75, and 76.67% following once, twice and thrice administrations, respectively (**Table 3**).

**Table 3:** The PTA of AUC/MIC for 1- or 1.5-log-unit decrease after intramammary administration in cows.

Dose Regimen	PTA (1-log decrease)	PTA (1.5-log decrease)
75 mg once	92.80	23.12
75 mg twice	97.14	60.75
75 mg thrice	97.94	76.67

## Discussion

*Staphylococcus aureus* is usually responsible for contagious mastitis transmitting between cows, as the uninfected quarters are normally exposed to the pathogen during milking (Gruet et al., 2001). Cefquinome is effective against *S. aureus* bovine IMI generally, considering that most pathogens are susceptible to this compound with low MIC value (Supplementary Table S2). The cefquinome MICs in this study are in line with the level of previous reports for bovine (Schmid and Thomas, 2002). Considerable activity of cefquinome has been reported against methicillin resistant *S. aureus* (MRSA) strains isolated from swine, chicken and even human (Aarestrup and Skov, 2010; Wang et al., 2014). MRSA strains were rarely isolated from clinical mastitis cases of bovine (Chin et al., 1992; Murphy et al., 1994). However, for pathogens isolated from human patients, the MICs were much higher ranging from 1 to 16 μg/mL and most of the isolates carried the mecA gene. Therefore, susceptibility distribution of cefquinome suggests that this drug has potential to treat the IMI caused even by either methicillin sensitive *S. aureus* (MSSA) or MRSA, which may largely benefit the general public health.

Histologically, the blood-milk barrier, formed by the close link of secretory cells at their apex by tight junctions in lactating udder, is also responsible for the passive transport of drug between both compartments, namely blood and milk (Gruet et al., 2001). These objective factors may explain the situation: when cefquinome is administered to only one gland, the drug concentration in the non-treated gland is as low as the LOQ (**Figure 2**) or not quantifiable. Comparable findings were reported in bovine in a previous study (Li et al., 2014). Therefore the influence of R4 drug concentration on L4 concentration or vice versa is negligible, and both fourth glands (R4 and L4) are employed and considered as independent study units for intramammary dosing.

After intramuscular or subcutaneous administration, the absorption of cefquinome is quick and complete into the circulation with a high bioavailability (Aarestrup and Skov, 2010; Zonca et al., 2011). Nevertheless, following intramammary infusion drug systemic absorption is limited, and drug concentration in blood is about 0.1 μg/mL in cow (Zonca et al., 2011; Li et al., 2014). Somehow, in our works, we found that drug concentration in blood is higher in mouse than in bovine (Yu et al., 2016). Considering the relative size of MG tissue, drug transportation from gland canal to blood may be easier to happen in mouse than in bovine. Even though, in our mouse study, the concentration in MG tissue is still much higher than in blood (Yu et al., 2016), which is in line with the cow blood/gland distribution pattern (Zonca et al., 2011). The observed very limited systemic absorption from localized MG dosing is likely because cefquinome, a β-lactam antibacterial and organic acid with pK_a_ of 2.51 ∼ 2.91, has limited lipid solubility to penetrate through membranes, such as the blood-MG barrier, due to its high degree of ionization in both plasma (pH = 7.4) and milk (pH = 6.5 ∼ 6.8). The long half-life of elimination (*t*_1/2-MG_) of 12.44 ± 0.81 and 12.66 ± 0.69 h calculated respectively using non-compartmental and one-compartmental models indicated that the concentration of cefquinome reached a very high level that was maintained for a very long time in MG tissue. However, the value of *t*_1/2-MG_ in mouse model is still somehow longer than that in bovine, which may be attributed to the variation between species and different composition of samples (generally milk samples from bovine). For the consideration of economic reasons, antibiotics should be selected and given with the shortest withdrawal period to make the milk qualified for marketing as soon as possible. Although the elimination of cefquinome from MG tissue was quite slow and the MRT was about 6–10 h, the residue of cefquinome in milk samples cannot be detected after 120 h following intramammary administration (Zonca et al., 2011; Li et al., 2014).

The killing activity of cefquinome in the current study is similar to the previous report, in which the first generation cephalosporins cefalexin, cefalonium, cefapirin, and cefazolin were investigated to treat the mouse *S. aureus* mastitis and a dramatic effectiveness was observed (Demon et al., 2012). Although a 5-log_10_CFU count is usually used as the initial inoculum *in vitro* killing trials, in this study a much higher bacterial load of 7.28 log_10_CFU/gland is employed in order to simulate an acute and severe IMI. Compared with the previous work (Yu et al., 2016), treatments of wild pathogens infection may call for a larger dose or more frequent dosing intervals, regardless of the fact that the MIC values are the same.

Even given the minimum dose of 25 μg/gland, the concentration of cefquinome in gland tissue during 24 h maintains over the MIC value, which provided a %T > MIC of 100% for all the dosing regimens tested in this study. For time-dependent drugs, antibacterial effectiveness is more closely linked to the exposed duration of bacteria than the concentrations, as long as the drug level is over MIC value. The 100% of %T > MIC means that the time required for killing activity is abundant during the entire observation period. In this situation, the correlation of %T > MIC versus the differences of bacterial counts (log_10_ CFU/gland) cannot be obtained for modeling purpose, suggesting that if a %T > MIC would be a preferred PK/PD driver, a much lower dose level might be needed. Under the current dosing schedules, AUC_0-24_/MIC is used instead to fit the data to the PK/PD models.

As indicated in **Figure 2**, the killing activity of cefquinome has elevated only slightly when the drug dose over 200 μg/gland, which suggests the regimens of 200, 400, and 800 μg/gland may be over dosed schedules in a mouse model. Similar results are observed in PK/PD sigmoid model (**Figure 3**) that identical decrease of colony counts may require quite different doses on the flat tail of the curve. Therefore, the AUC/MIC indices achieving a 1- and 1.5-log-unit decrease at the knee points were used as the target value in the Monte Carlo simulation, which were 4714.72 and 16571.55 h⋅mL/g, respectively. The recommended dose regimen of cefquinome for treatment of bovine mastitis was three infusion of 75 mg per mammary quarter (The European Agency for the Evaluation of Medicinal Products Veterinary Medicines and Inspections, 1998), and the PTAs of 75 mg administration for once, twice, and thrice were estimated. However, narrow frequency distribution of MIC is a considerable limit of the Monte Carlo simulation, as the susceptibilities to cefquinome are mainly concentrated in 0.25–0.5 μg/ml of this population. According to the previous papers, MIC levels of cefquinome or ceftiofur, against *S. aureus* (either mastitis isolates or not), are varied from 0.25 to 1 μg/ml but mainly distribute at 0.5 μg/ml (Zonca et al., 2011; Oliveira et al., 2012; Wang et al., 2014), which are similar to our report. These findings suggested that at most 76.67% infected mammary quarter could be cured, but not bacterially eradicated.

In summary, our study indicates that *in vivo* analysis of antimicrobials is of utmost importance to improve their therapeutic potential. This is the first study ever to assess glandular tissue PK/PD integration for investigating the effectiveness of cefquinome. Additionally, our data highlight the impact of anatomical structure (blood-milk barrier) on the drug distribution and PK characteristics in blood and gland tissue compartments. The glandular tissue PK/PD simulation demonstrates that the value of %T > MIC is generally 100%, the maximum limit in PK/PD principle, following an intramammary infusion administration. Instead, the AUC_0-24_/MIC serves as substitute parameters under these particular conditions of drug, microbe and local inflammation combination. The magnitude of PK/PD parameters to achieve a remarkable antibacterial efficacy is assessed in this study in relation to treat IMI. The clinical recommended therapeutic regimen can achieve approximately 76.67% cure rate as predicted by Monte Carlo simulation.

## Author Contributions

Y-HL conceived of the study and given the final approval of the version to be published. YY participated in design of the study and drafted the manuscript. Y-FZ carried out the pharmacokinetic studies. XL and M-RC carried out the animal experiments of pharmacodynamic work. JS and X-PL have made substantial contribution to analysis and interpretation of data. G-LQ has been involved in revising the manuscript critically for important intellectual content. All authors read and approved the final manuscript.

## Conflict of Interest Statement

The authors declare that the research was conducted in the absence of any commercial or financial relationships that could be construed as a potential conflict of interest.

## Supplementary Material

FIGURE S1 ***In vitro* time-killing curves of cefquinome against *S. aureus* isolates with initial bacterial load of 7-log-unit**.

FIGURE S2 **The concentration-time curves of both glands, following cefquinome administration into only one MG.** The level of cefquinome in the non-treated gland was over 100 times lower than in the administrated one and as low as the LOQ, suggesting that the influence of drug administration to one gland on the concentration of the other non-dosed side should be negligible in the same subject.
