# Supplementary material for: Dose Assessment of Cefquinome by Pharmacokinetic/Pharmacodynamic Modeling in Mouse Model of Staphylococcus aureus Mastitis
Source: Front Microbiol. 2016 Oct 7;7:1595. doi: 10.3389/fmicb.2016.01595 (PMC5053985; doi:10.3389/fmicb.2016.01595)
Supplement: Supplementary file 1 [file Table_1.DOCX]

Supplementary Material

**Dose assessment of Cefquinome by PK/PD Modeling in Mouse Model of *Staphylococcus aureus* Mastitis**

Yang Yu^1,2^, Yu-Feng Zhou^1,2^, Xiao Li^1,2^, Mei-Ren Chen^1,2^, Gui-Lin Qiao^3^, Jian Sun^1,2^, Xiao-Ping Liao^1,2^, Ya-Hong Liu^1,2^**^＊^**

*** Correspondence:** Dr. Ya-Hong Liu Email: [lyh@scau.edu.cn](mailto:lyh@scau.edu.cn)

1. **Supplementary Tables**

**Table S1.** The extraction recovery of CEQ and coefficient of variation of intra-assay and inter-assay in mammary gland tissue.

| Concentration (μg/gland) | R_E_ (%) | Intra-assay  CV (%) | Inter-assay  CV (%) |
| --- | --- | --- | --- |
| 10 | 95.43 ± 2.16 | 2.98 | 2.26 |
| 20 | 87.86 ± 4.99 | 2.77 | 7.06 |
| 50 | 72.58 ± 3.22 | 3.65 | 8.21 |
