# Supplementary material for: Dose Assessment of Cefquinome by Pharmacokinetic/Pharmacodynamic Modeling in Mouse Model of Staphylococcus aureus Mastitis
Source: Front Microbiol. 2016 Oct 7;7:1595. doi: 10.3389/fmicb.2016.01595 (PMC5053985; doi:10.3389/fmicb.2016.01595)
Supplement: Supplementary file 2 [file Table_2.DOCX]

Supplementary Material

**Dose assessment of Cefquinome by PK/PD Modeling in Mouse Model of *Staphylococcus aureus* Mastitis**

Yang Yu^1,2^, Yu-Feng Zhou^1,2^, Xiao Li^1,2^, Mei-Ren Chen^1,2^, Gui-Lin Qiao^3^, Jian Sun^1,2^, Xiao-Ping Liao^1,2^, Ya-Hong Liu^1,2^**^＊^**

*** Correspondence:** Dr. Ya-Hong Liu Email: [lyh@scau.edu.cn](mailto:lyh@scau.edu.cn)

1. **Supplementary Tables**

**Table S2.** The susceptibility of CEQ against *S. aureus*.

| Sample No. | Source | MIC range (μg/mL) | MIC_90_ (μg/mL) | Reference |
| --- | --- | --- | --- | --- |
| MSSA |  |  |  |  |
| JP41 | Cow mastitis | 0.5 | - | Present study |
| ATCC 29213 | - | 0.25 | - | ([Wang et al., 2014](#_ENREF_17)) |
| 38 | Cow mastitis | 0.25-0.5 | 0.5 | ([Yu et al., 2016](#_ENREF_19)) |
| 20 | Human | 0.25-0.5 | 0.5 | ([Murphy et al., 1994](#_ENREF_10)) |
| 20 | Human | 0.5-4 | 2 | ([Chin et al., 1992](#_ENREF_4)) |
| 98 | Patients | 0.25-4 | 2 | ([Aarestrup and Skov, 2010](#_ENREF_1)) |
| 11 | Horse / Foal | 0.25-0.5 | 0.5 | ([Thomas et al., 2006](#_ENREF_16)) |
| MRSA |  |  |  |  |
| 2 | Chicken / Pork | 0.5 | - | ([Wang et al., 2014](#_ENREF_17)) |
| 10 | Human | 1- >16 | 16 | ([Murphy et al., 1994](#_ENREF_10)) |
| 20 | Human | 1-16 | 8 | ([Chin et al., 1992](#_ENREF_4)) |
| 71 | Human / Pig | 1-16 | 4 | ([Aarestrup and Skov, 2010](#_ENREF_1)) |
