# Supplementary figures and images for: Dose Assessment of Cefquinome by Pharmacokinetic/Pharmacodynamic Modeling in Mouse Model of Staphylococcus aureus Mastitis
Source: Front Microbiol. 2016 Oct 7;7:1595. doi: 10.3389/fmicb.2016.01595 (PMC5053985; doi:10.3389/fmicb.2016.01595)

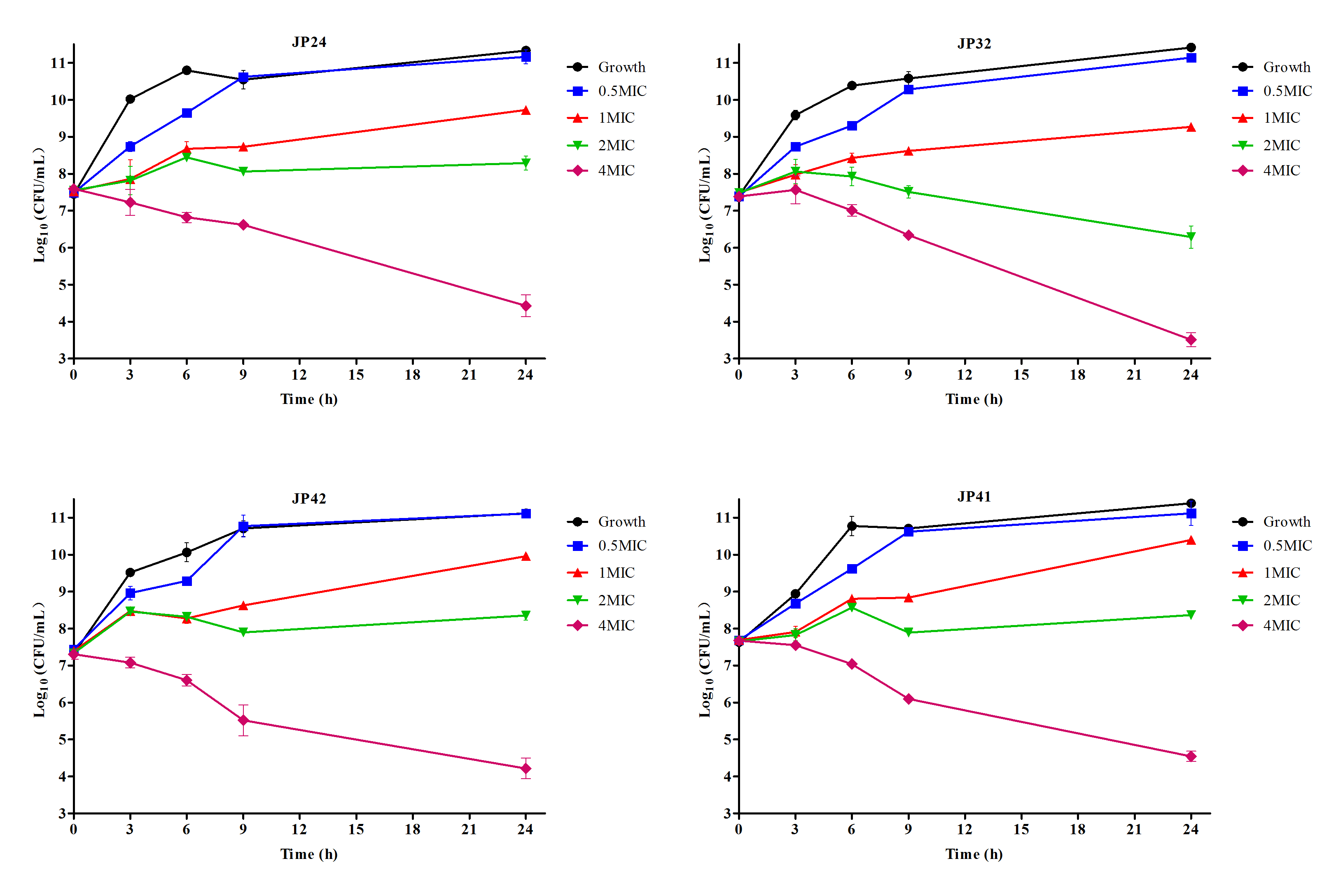

Supplement: FIGURE S1 — In vitro time-killing curves of cefquinome against S. aureus isolates with initial bacterial load of 7-log-unit. [file Image_1.TIF]

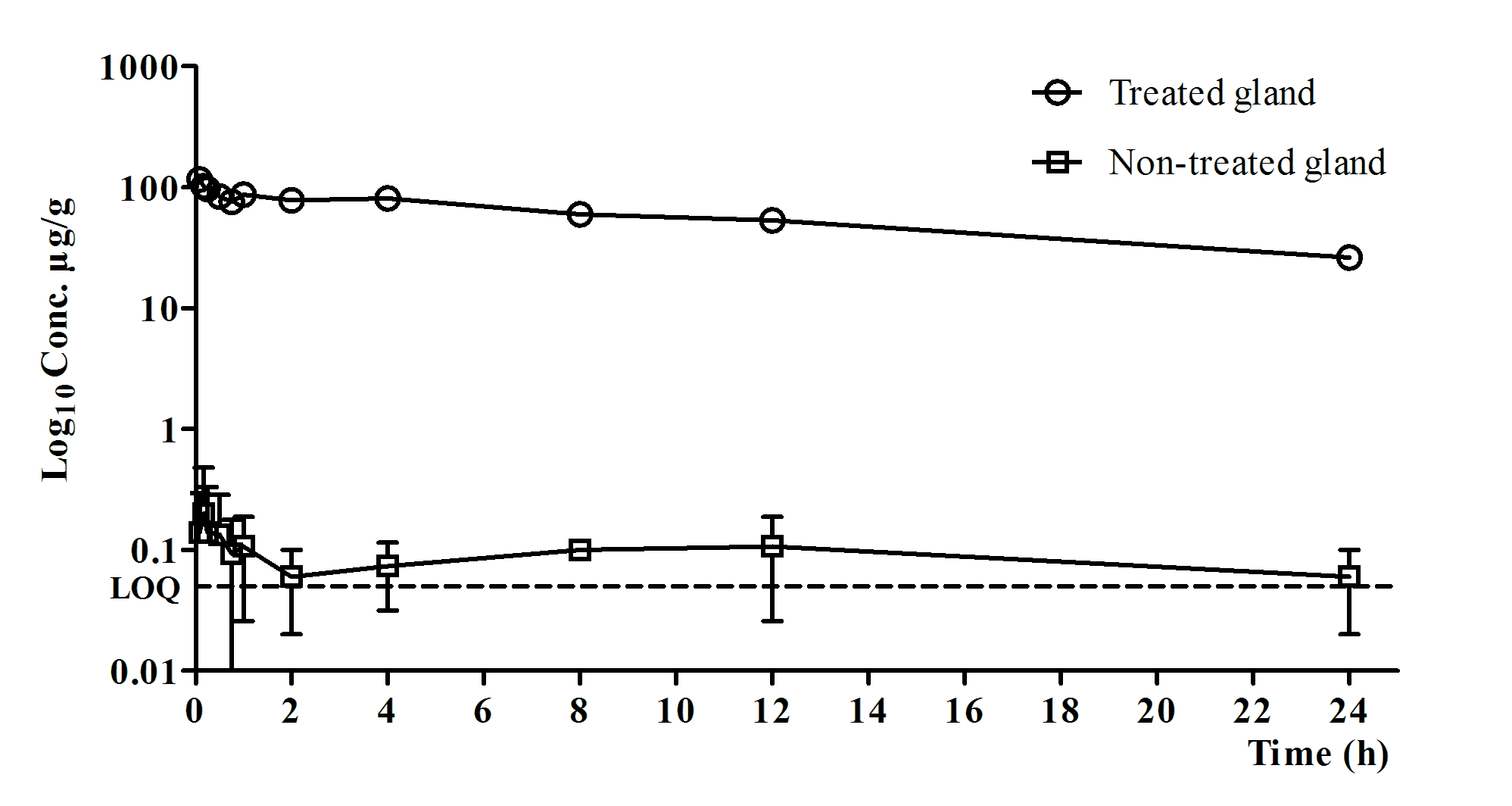

Supplement: FIGURE S2 — The concentration-time curves of both glands, following cefquinome administration into only one MG. The level of cefquinome in the non-treated gland was over 100 times lower than in the administrated one and as low as the LOQ, suggesting that the influence of drug administration to one gland on the concentration of the other non-dosed side should be negligible in the same subject. [file Image_2.TIF]
